# A quantitative method to assess muscle edema using short TI inversion recovery MRI

**DOI:** 10.1038/s41598-020-64287-8

**Published:** 2020-04-29

**Authors:** Julia R. Dahlqvist, Ruth Salim, Carsten Thomsen, John Vissing

**Affiliations:** 1 Copenhagen Neuromuscular Center, Section 3342, Department of Neurology, Rigshospitalet, University of Copenhagen, Blegdamsvej 9, 2100 Copenhagen, Denmark; 2 Department of Radiology, Rigshospitalet, University of Copenhagen, Blegdamsvej 9, 2100 Copenhagen, Denmark

**Keywords:** Neuromuscular disease, Inflammation

## Abstract

Muscle inflammation is an important component of disease pathophysiology in several muscular dystrophies. Hyperintensities on MRI sequences with short TI inversion recovery (STIR) reflect edema, or inflammation (STIR+). Conventionally, STIR evaluation has been done by visual inspection. In this study, we developed a quantitative STIR method, and tested its ability to identify STIR+ lesions in healthy controls and patients with Facioscapulohumeral muscular dystrophy and compared the results with visual STIR evaluation and quantitative T2 relaxation time mapping. The method was based on pixel-by-pixel histograms of the distribution of signal intensities from muscles. Signal intensities from healthy control muscles were averaged and used to define an upper reference limit. Muscles with >2.5% pixels above the limit were defined as being STIR+. The new method showed agreement with T2 relaxation time mapping in 95% of muscles. The visual STIR method only showed agreement with the quantitative STIR method and T2 relaxation time mapping in 88 and 84%, respectively. STIR sequences are available on most MR scanners and the post-processing used in the new quantitative method can be performed using free software. We therefore believe that the new method can play an important role in identifying STIR+ lesions in patients with neuromuscular diseases.

## Introduction

Muscle inflammation is likely an important component of disease pathophysiology, not just in primary inflammatory myopathies, but also in several muscular dystrophies^1^. For the same reason, steroid treatment in inflammatory myopathies and Duchenne muscular dystrophy is a mainstay, and other immunomodulatory treatments are under investigation in several muscular dystrophies^2–4^.

Histological examination of muscle tissue can identify inflammation and describe its cellular aspects. However, it is invasive and only able to evaluate tiny fractions of a single muscle at a time. Skeletal muscle MRI is non-invasive and can investigate multiple muscles simultaneously. Sequences with short TI inversion recovery (STIR) are designed to suppress fat signal and enhance signal from tissues with long T1 and T2 relaxation times, such as inflammatory tissue. Hyperintense lesions on STIR images (STIR+ lesions) have therefore been used as a marker of muscle inflammation in muscle diseases^5–7^. STIR+ lesions may reflect hydrostatic edema, sarcolemma leakiness after for instance exercise, and necrosis, and is therefore not a marker which is as specific as a muscle biopsy in demonstrating inflammation. However, biopsies from STIR+ lesions in patients with muscle disease have repeatedly demonstrated inflammatory changes when such lesions have been biopsied^8,9^, as well as upregulated genes involved in inflammatory pathways^8^.

When assessing STIR sequences, this is conventionally done in a qualitative way by visual inspection, where the contrast in signal intensity between muscles are used to determine if a muscle is STIR+. If all muscles in a segment are inflamed, then likely such a muscle would be deemed not to be STIR+. By contrast, if almost all muscles in a segment are replaced by fat, then a preserved muscle with low fat content, but surrounded by fat-replaced muscle tissue, will look STIR+, although it is not. Because of this, and since evaluation of muscle inflammation might be important in prediction of prognosis and intervention effects, there is a need for quantitative assessment of muscle edema/inflammation. T2 relaxation time mapping is a quantitative MRI method that indicates muscle edema, or inflammation, comparable to hyperintensities on STIR images^1^. The data processing of T2 relaxation time mapping is, however, relatively time consuming and the water T2-time can be affected by high muscle fat content, which is frequently found in patients with muscular dystrophies.

In this study, we aimed at testing whether assessment of STIR+ lesions, which are imprecise on visual inspection, can be made more quantitative/precise by using phantoms as comparators. We have developed a quantitative way of assessing STIR, and have tested this new method’s ability to identify STIR+ lesions in healthy controls and patients with Facioscapulohumeral muscular dystrophy (FSHD) and compared the results with conventional visual STIR evaluation and quantitative T2 relaxation time mapping of muscles.

## Materials and Methods

### Ethical approval

All participants gave written informed consent to participate and the study was conducted in accordance with the declaration of Helsinki. The study was approved by the Danish National Committee on Health Research Ethics (approval numbers: H-15009760; H-18023049).

### Study design and subjects

We recruited healthy controls from the local community and patients with genetically verified FSHD type 1 from our clinic. D4Z4 repeats were determined by Southern blotting and all patients included had less than 10 D4Z4 repeats in leukocytes DNA. Inclusion criteria for patients were (i) age over 18 years and (ii) STIR+ lesion in at least one muscle on MRI. All examinations were performed from November 2018 to January 2019.

### MR imaging

All participants were scanned using a 3.0T Siemens scanner (MAGNETOM Verio Tim System; Siemens AG, Erlangen, Germany). All participants were placed supine in a feet first position with body matrix coils on top of thighs and calves. Sealed calibration tubes were placed along the legs in the same individual order. These tubes were filled with Gadovist® (0.55 mmol/L) and the content was left unchanged throughout the duration of the entire study. The fluid was chosen because of its signal characteristics and used to calculate a calibration factor for each examination. The calibration factor was used to normalize the signal intensities and enable comparisons across examinations. The MRI protocol for thighs and calves included localizers, T1-weighted images, 2-point Dixon, STIR, and T2-weighted multi-echo images. The technical settings were: T1-weighted images, slice thickness = 3.0 mm, distance facto r = 100%, echo time (TE)/repetition time (TR) = 39/656 ms; Dixon images, slice thickness = 2.0 mm, distance factor = 20%, TE/TR = 2.45 and 3.675/5.64 ms; STIR images, slice thickness 6.0 mm, distance factor = 100%, TE/TR = 29/3000 ms, inversion time = 230 ms; T2-weighted multi-echo images (17 images), slice thickness 10.0 mm, distance factor = 260%, TE = 9, 18, 27, 36, 45, 54, 63, 72, 81, 90, 99, 108, 117, 126, 135, 144, and 153 ms, TR = 2870 ms. For T1-weighted, Dixon, and STIR sequences, the field of view was 450 mm and for the T2-weighted multi-echo sequence, it was 200 mm. Three patients with FSHD were also examined with proton MR spectroscopy. The volume selective proton MRS PRESS was used with the TEs 33, 40, 48, 57, 68, 82, 99, 118, 142, and 170 ms. The volumes of interest were fitted for the individual muscle and ranged from 10 mm × 10 mm × 15 mm to 20 mm × 20 mm × 20 mm. In the healthy controls, only the right leg was scanned. The entire protocol was completed within 60 minutes.

### MRI analysis

#### Muscle fat fractions

We mapped all thigh muscles manually on Dixon images at the slice corresponding to 50% of the length of femur and calf muscles at 33% of the length of tibia. Muscle fat content was calculated by expressing the fat signal as a percentage of the total water and fat signal in an advanced model where we compensated for T1 and T2* relaxation effects according to Buxton *et al*.^10^ using fixed values for T1_muscle_ (1420 ms), T1_fat_ (371 ms), T2*_fat_ (133 ms), and T2*_muscle_ (31.8 ms)^11^. We also adjusted for the spectral complexity of lipid molecules according to Zhong *et al*.^12^. The precision of the manual mapping has previously been established (R = 0.976, P < 0.0001, n = 80)^13^.

#### STIR signal intensities

All muscles were evaluated visually on the STIR sequences by J.R.D. and J.V., assessing the presence or absence of signal hyperintensity in each muscle according to the routine standard of reviewing STIR images.

We transferred the muscle mapping from Dixon to STIR images for quantification of edema. We registered mean signal intensity of all muscles and performed an analysis of pixel by pixel histograms. Pixel by pixel histograms of the distribution of signal intensities from healthy control muscles were averaged and used to define an upper reference limit. The signal intensities of the healthy control muscles were normally distributed and the upper reference limit was therefore set at mean + 2 standard deviations (SD). Pixels with signal intensities greater than this reference limit were defined to have elevated signal intensity, and the number of pixels with elevated signal intensity was expressed as a percentage of total pixels in each mapped muscle. Muscles with less than 2.5% elevated pixels were defined as being quantitatively STIR negative (qSTIR−). Muscles with more than 2.5% elevated pixels were defined as being quantitatively STIR positive (qSTIR+). The cut-off value of 2.5% was based on the fact that, in a normal distribution, 95% of values lie within 2 SD of the mean, 2.5% in a lower tail, and 2.5% in an upper tail.

STIR is relatively insensitive to B1 and B0 inhomogeneities^14^, which can increase noise. To counteract this, we used the TrueForm^TM^ Technology with significant improvement in B1 homogeneity and performed bias shimming and used a pre-scan receiver coil B1 filter in all STIR acquisitions. Signal intensities measured in the calibration tubes were used to calculate a calibration factor per examination that was used to linearly scale all intensities relative to the background:

$$C{F}_{pe}={S}_{CT}/{S}_{mean}$$

where CF_pe_ is the calibration factor per examination, S_CT_ the signal intensity of the calibration tube and S_mean_ the mean signal intensity of all calibration tubes. The mean signal intensities of mapped muscles and the upper reference limit were then divided by the calibration factor per examination. Image intensity non-uniformity variation was corrected by using signal intensities from the subcutaneous fat. The leg was divided into slices like pieces in a pie chart. The signal intensity from the subcutaneous fat in the same pie slice as the muscle was compared with fat in the slice nearest to the calibration tube and corrected.

One trained evaluator performed all analyses using Osirix MD software.

#### T2 relaxation times

Finally, we calculated the water T2 relaxation time of muscles. The muscle mapping was again transferred from the Dixon images. The T2 relaxation time mapping was based on a two-component model where muscle and fat were used in a bi-exponential curve fit to estimate muscle T2_water_, T2_fat_ and fat fraction. The method was similar to the method described by Azzabou *et al*.^15^. However, we did not adjust for B1 variations. We used 90° and 180° pulses and Carr-Purcell-Meiboom-Gill refocusing to minimize stimulated echoes. Since the Carr-Purcell-Meiboom-Gill sequence compensates for flip angles in the 180° pulses for the even echoes, the first echo (TE = 9 ms) was excluded from the calculations.

Before using the model on our data, we performed simulations. The simulations showed that the model had the constrains that T2_fat_ should be below 150 ms. Previous studies have found T2_fat_ of 133 ± 4.43 ms^16^. The T2_fat_ measured in the subcutaneous tissue of our patients was 140.1 ± 9.8 ms (range 129.3–150.3 ms). We therefore constrained the T2_fat_ in the bi-exponential fit to a maximum of 150 ms.

MRS was used to test the bi-exponential described above. We examined fifteen muscles in three patients with FSHD. Spectra were sampled in 4–6 different calf muscles in each of the three patients: the medial gastrocnemius (N = 5), lateral gastrocnemius (N = 4), peroneus (N = 3), anterior tibialis (N = 2), and posterior tibialis muscles (N = 1). At the exact same location as the volume of interest, a volume of the same size was mapped at a T2-weighted image and the muscle T2_water_ was calculated using the bi-exponential model described above. Muscle T2_water_ calculated using the bi-exponential model increased with increasing T2_water_ determined by MRS (r = 0.64; p = 0.01). The mean difference between the two methods was −0.7 ± 4.0 ms (MRS minus bi-exponential results).

The muscle T2_water_ of the healthy control muscles were normally distributed and the upper reference limit was therefore set at mean + 2 SD.

### Statistical analyses

Statistical analysis was performed using SPSS v25 and values are mean ± SD unless otherwise stated. We used Pearson correlation to test relations between parameters. A p-value of <0.05 was considered significant.

## Results

We included 9 healthy controls and 7 patients with FSHD (Table 1). Nine thigh and 6 calf muscles were analyzed in each leg. The STIR images of the calves from one healthy control had artefacts over the calibration tube which made the calibration of signal intensities impossible and the analysis incomparable to the other scans. They were therefore excluded. Further, one end-stage FSHD muscle, where the muscle was totally replaced by fat, was excluded due to difficulties mapping the muscle. This resulted in 258 included control muscles and 209 FSHD muscles in the analyses of muscle fat fraction and STIR.

**Table 1:** Demographics of participants.

	Healthy controls	Patients with FSHD	P-value
Sex, M/F	6/3	5/2	
Age, years	49.8 ± 9.7	49.3 ± 9.2	0.46
Mean muscle fat fraction, %	8.7 ± 4.6	39.1 ± 27.9	<0.0001

M = male; F = female; FSHD = Facioscapulohumeral muscular dystrophy; P-value indicates whether the group of healthy controls and the group of patients with FSHD were significantly different.

### Dixon method

Muscle fat fraction was 8.7 ± 4.6% in healthy controls and 39.1 ± 27.9% in patients with FSHD calculated by the advanced model correcting for T1, T2* and 8 fat components. Fat fractions calculated by the advanced model correlated well with fat fractions calculated by the bi-exponential model (T2 relaxation time mapping) (R = 0.99; p < 0.0001).

### STIR analyses

When visually evaluating all STIR sequences, we identified 6 healthy control muscles and 32 FSHD muscles that were STIR+. The STIR+ healthy control muscles were not included when defining the upper reference limit used in the quantitative STIR analysis. The adjusted mean STIR signal intensity of all STIR− healthy control muscles was 110.6 ± 38.2 and the calculated upper reference limit 187 (mean + 2 SD). This limit was then individualized per examination based on the calibration factor.

Using the quantitative STIR method, we identified all muscles with more than 2.5% elevated pixels. We found 12 qSTIR+ muscles in the healthy controls and 41 in the patients with FSHD. The qualitative and quantitative methods showed agreement in 88% of all analyzed muscles (Table 2). While only 3% of the healthy muscles showed disagreement, 22% of FSHD muscles showed disagreement.

**Table 2:** Quantitative STIR vs. visual evaluation on STIR images and T2 relaxation time mapping.

			Quantitative STIR		Total
			qSTIR−	qSTIR+	
Visual STIR	STIR−	Healthy control	245	7	252
		FSHD	149	28	177
		*Total*	394	35	429
	STIR+	Healthy control	1	5	6
		FSHD	19	13	32
		*Total*	20	18	38
Total			414	53	467
T2-time	<41 ms	Healthy control	123	1	124
		FSHD	74	2	76
		*Total*	197	3	200
	> 41 ms	Healthy control	3	3	5
		FSHD	7	28	35
		*Total*	10	31	40
Total			206	34	240

(q)STIR− = (quantitative) STIR-negative muscle; (q) STIR+ = (quantitative) STIR-positive muscle; FSHD = Facioscapulohumeral muscular dystrophy

### T2 relaxation times

In patients with FSHD, we determined the T2 relaxation times in muscles with fat fractions below 50% to minimize the effect of fat (N = 111). In the healthy controls, we only scanned and analyzed the right thigh and calf (N = 129).

The mean muscle T2_water_ for the healthy muscles was 33.9 ± 3.5 ms. We used this value to define a cut-off for normal muscle T2_water_ and set it at 41 ms (mean + 2 SD). We then compared the quantitative STIR method with the T2 relaxation time mapping (Table 2). In 95% of the muscles, the two methods showed agreement. Compared to visual STIR evaluation, T2 relaxation time mapping showed agreement in 84% of the muscles.

**Figure 1:**
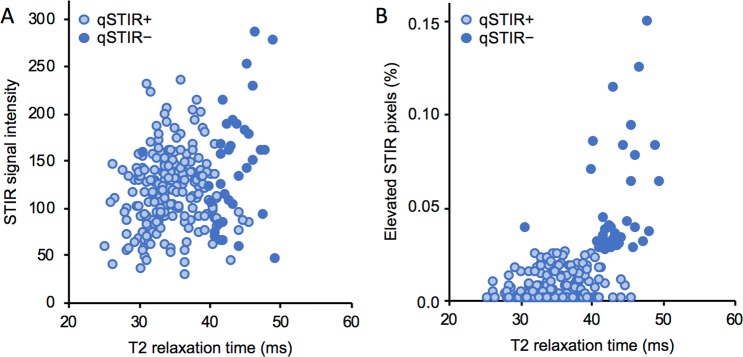
The relation between quantitative STIR and T2 relaxation time mapping of muscle. The linear regression between the T2 relaxation times and STIR signal intensities (**A**), and percentage of elevated STIR pixels (**B**) of healthy and dystrophic skeletal muscles. The dashed line in figure A indicates the cut-off of normal T2-time. qSTIR− = quantitative STIR-negative muscle; q STIR+ = quantitative STIR-positive muscle.

**Figure 2:**
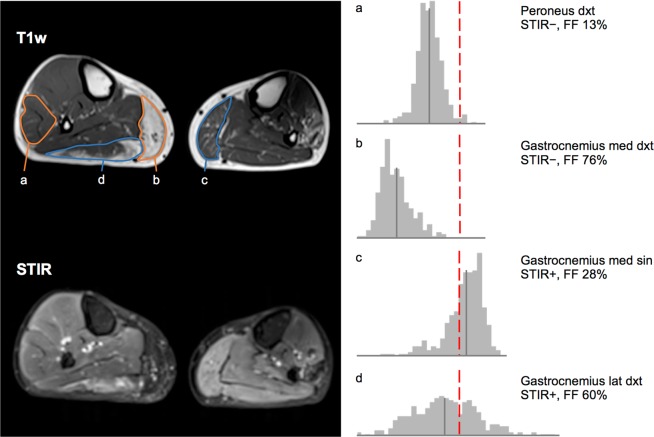
STIR sequences of muscles. T1-weighted (T1w) and STIR images of calf muscles in a patient with FSHD. Pixel by pixel histograms of the distribution of STIR signal intensities of four muscles are shown to the right. Their mean signal intensity is marked by a filled gray line in each histogram. The right peroneus muscle (**a**) is STIR− and has normal fat fraction. The histogram of all signal intensities is normally distributed. The right gastrocnemius medialis (**b**) is also STIR− but is severely fat replaced. The histogram is negatively skewed and the mean signal intensity is correspondingly lower. The left gastrocnemius medialis (**c)** is STIR+ and has normal fat fraction. Compared to muscle (**a**), the histogram is positively skewed resulting in an increased mean signal intensity that corresponds to the higher water fraction. The right gastrocnemius lateralis (**d**) is also STIR+ but is severely fat replaced. The combination of high water and fat content results in mean signal intensity similar to muscle (**a**). The percentage of pixels above the upper reference limit (red dashed line) better corresponds with the water content.

There was a weak positive correlation between the adjusted STIR signal intensities and the muscle T2_water_ (R = 0.27; p < 0.0001; Fig. 1A). However, as illustrated by the color of the circles in Fig. 1A, there was a great variation in the adjusted mean STIR signal intensity of muscles that were qSTIR+ and/or had T2-elevation. This disassociation is probably partly explained by the high fat content in dystrophic muscle. STIR sequences are designed to suppress fat and high muscle fat content therefore result in lower mean signal intensity (Fig. 2). There was a stronger correlation between the percentage of elevated STIR pixels and the T2 relaxation times (R = 0.58, p < 0.0001; Fig. 1B).

### Test-retest of STIR

**Figure 3:**
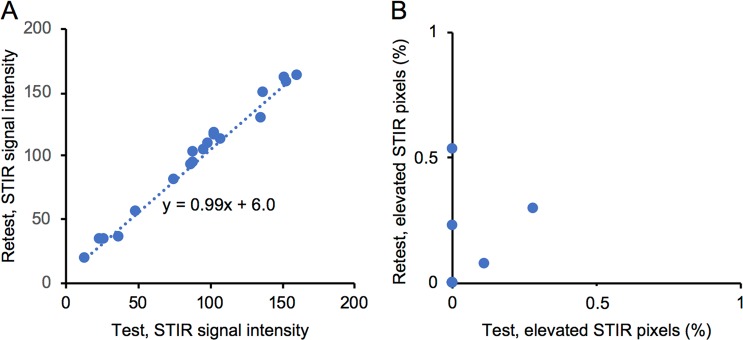
Test-retest of STIR signal intensity. The linear regression between two MR scans of the same participant. (**A**) The adjusted STIR signal intensities of muscles and (**B**) the percentage of elevated STIR pixels. The dashed line in A is the linear regression line (y = 0.99x + 6.0).

To give an estimate of the variation in signal intensity from one scan to another, we performed two scans in one day in one of the healthy controls. The adjusted STIR signal intensities from the two scans correlated strongly (R = 0.99, p < 0.0001; Fig. 3A). Since all muscles were qSTIR−, there was a floor effect, and the percentage of elevated pixels did not correlate.

## Discussion

In this study, we evaluated if quantitative STIR could identify STIR+ lesions in skeletal muscle in healthy controls and patients with FSHD. We compared the results from the quantitative STIR method with results from visual evaluation of the muscles on STIR images and quantitative T2 relaxation time mapping. We found that the quantitative STIR method showed agreement with T2 relaxation time mapping in 95% of the muscles. Visual evaluation of STIR sequences only showed agreement with quantitative STIR and T2 relaxation time mapping in 84–88% of the muscles. STIR sequences are available on most MR scanners and the post-processing used in the new quantitative method can be performed using free imaging software. We therefore believe that the new method can play an important role in identifying STIR+ lesions in muscles of patients with neuromuscular diseases.

Visual inspection of STIR MR imaging has routinely been used to evaluate hyperintensities in skeletal muscle as an indicator of inflammation in a variety of neuromuscular diseases^5,6^. However, quantitative assessment is generally superior to qualitative visual inspection, as quantitative methods are observer-independent and can report changes on a continuous scale versus an ordinal scale for visual inspection^17^. This has been shown when investigating muscle fat content, where the quantitative Dixon MRI detect changes in progression of fat replacement that visual evaluation cannot see^13,17,18^. Quantitative Dixon MRI is more precise and reliable than visual methods for evaluation of fat fractions for longitudinal follow-up or therapy evaluation^19^. We therefore developed a method to quantitatively identify STIR+ lesions on STIR sequences, by measuring elevated pixels in regions of interest and calibrating the intensities using a calibrating tube at each scan. We identified qSTIR+ lesions in 19.6% (N = 41) of FSHD leg muscles. Thirty of these qSTIR+ muscles were included in the T2 relaxation time mapping and 28 showed T2-elevation. Comparing qualitative and quantitative STIR analyses, only 31.7% of the qSTIR+ muscles were visually evaluated as being STIR+, and conversely, of the 32 FSHD muscles that were visually evaluated as STIR+, only 13 were quantitatively qSTIR+. Many of the muscles that were visually STIR+ but qSTIR− were surrounded by muscles with high muscle fat content or had many vessels, which could explain the erroneous assumption that these sites were STIR+, which illustrates the weakness of sole visual STIR inspection. These results emphasize that quantitative STIR is a much stronger tool to assess muscle edema than the usual qualitative STIR evaluation by visual inspection.

Upgrading conventional STIR images to a quantitative methodology has been attempted before^7,20,21^. To get around the confounding factors that may affect the STIR signal intensities, such as field inhomogeneity and location-dependent signal variation, previous studies used signal intensity ratios instead of absolute signal intensities^7,20,21^. The ratio of the signal intensity of a muscle of interest to a reference region (a healthy muscle, white brain matter, bone marrow, or calibration tubes) was then compared. STIR muscle signal intensity ratios were able to monitor motor nerve regeneration and predict functional recovery after complete traumatic transection of a forearm nerve^20^. In patients with thyroid-associated eye disease, there was higher STIR signal intensity ratios of extraocular muscles in patients with active disease compared to both patients with inactive disease and healthy controls^21^. In patients with FSHD, Janssen *et al*. used signal intensity from bone marrow to normalize the STIR muscle signal intensities and identified qSTIR+ lesions in 4.3% of leg muscles^7^. In our study, we identified almost 5 times as many qSTIR+ muscles (19.6%), which is partly explained by our inclusion criteria, dictating that patients had to have at least one STIR+ muscle on MRI to be included. It might also be explained by the different methodology used. Instead of STIR muscle signal intensity ratios, we used percentage of elevated pixels. In muscles with high fat content, the histogram of the signal intensities of all pixels is negatively skewed due to the suppression of fat tissue in STIR sequences resulting in a lower mean signal intensity. The “fat pixels” do not affect the percentage of elevated pixels, however. We were therefore able to identify more STIR+ muscles. We also confirmed the STIR-positivity by T2 relaxation time mapping, which they did not.

T2 relaxation time mapping is a quantitative MRI method that can act as an indicator of disease activity in myositis, Duchenne muscular dystrophy, and FSHD^1,22,23^. Elevated T2-time indicates muscle edema/inflammation and correspond to hyperintensities found on STIR images. In our study, we compared the identification of STIR+ muscles using the new quantitative STIR method with elevation of T2 relaxation time and found agreement in 228 out of 240 muscles.

We found that there was a positive correlation between the adjusted STIR signal intensity and the T2-time (R = 0.27). Das *et al*. also investigated the relation between normalized STIR signal intensity and T2 relaxation time in the eye muscles of 62 patients with thyroid eye disease and 6 healthy controls^24^. They found that there was a positive correlation between the two measures (R = 0.42). The correlation in that study, as well as in ours, was thus not convincing. Some of the disassociation between adjusted STIR signal intensity and T2 relaxation time can probably be explained by the different fat content in muscles. High fat content has a negative effect on the STIR signal intensity. In our study, we therefore investigated the percentage of elevated STIR pixels. The percentage of elevated STIR pixels showed a stronger correlation with muscle T2_water_ (R = 0.58) than the mean STIR signal intensity. The discrepancy, still present, between the percentage of elevated STIR pixels and muscle T2_water_ may partly be caused by the manual mapping. Especially in muscles with small cross-sectional areas and a small number of total pixels, unintentional inclusion of pixels from surrounding vessels or fat tissue can affect the results in a positive or negative direction, respectively. Also, the slice positions of the STIR and T2-weighted sequences in our study were not always identical and varied by up to 1 cm. Small vessels that had a course that was cross-sectional to the slice orientation in one slice could have a course longitudinal to the slice orientation in another slice and, thus, increase the signal intensity in the second slice.

In conclusion, we showed that quantitative STIR can identify qSTIR+ lesions with the same accuracy as T2 relaxation time mapping. STIR sequences are easily accessible on most MR scanners and the post-processing used in the new method was less time consuming than the T2 relaxation time mapping, especially once the upper reference limit had been determined. We therefore believe that this new method can be used to identify STIR+ lesions in patients with neuromuscular diseases.

## Data Availability

The datasets generated during and/or analysed during the current study are available from the corresponding author on reasonable request.
